# Assessment of Medical Professionalism among Students and Faculty Members of Shalamar Medical and Dental College, Lahore

**DOI:** 10.30476/JAMP.2021.88433.1342

**Published:** 2021-10

**Authors:** SHAZIA RASUL, M. ZAHID BASHIR, SAROSH SALEEM, SHABNAM TAHIR, AFLAK RASHEED, MUHAMMAD ALI SABIR

**Affiliations:** 1 Shalamar Medical & Dental College (SMDC), Lahore, Pakistan; 2 Federal Post graduate Medical Institute Sheikh Zayed Hospital, Lahore, Pakistan; 3 Shalamar Hospital, Lahore, Pakistan

**Keywords:** Assessment, Medical professionalism, Medical students, Faculty

## Abstract

**Introduction:**

Medical professionalism is an essential part of training and professional development of medical students. Unprofessional behavior in medical school may lead to professional misconduct in the future careers. The Learner’s Attitude of Medical Professionalism Scale (LAMPS) is a self-assessment questionnaire. It has been used in this study to assess and compare self-reported attitudes about different domains of medical professionalism among First and Final Year students and Faculty of Shalamar Medical and Dental College (SMDC). LAMPS has been used to identify the gaps in the attitudes of medical students and professionals, which can be addressed through a training program of professionalism.

**Methods:**

This is a cross-sectional survey conducted in SMDC from June to Dec 2018. First Year and Final Year Students and Faculty were recruited by non-probability convenience sampling. The sample size was calculated by the Cochran’s Formula, keeping the level of significance at 5% and margin of error at 3%. The reliability of LAMPS using Cronbach’s alpha is 0.7. It has been validated by 32 experts followed by pilot testing. The domains of professionalism were scored according to Likert Scale. The data were analyzed using SPSS 24. T-test was used for comparison of the means.

**Results:**

There were a total of 204 study participants; 88 students from First Year, 78 from Final Year and 38 Faculty Members. Honor/Integrity was the most valued trait and Excellence/Autonomy was the lowest scored domain of medical professionalism. There was a significant difference among attitudes of the First and Final Year students in the domains of Excellence/Autonomy and Altruism. Excellence/Autonomy and Honor/Integrity showed a significant difference between the Final Year students and Faculty.

**Conclusions:**

Medical students and faculty have significantly different views of certain attributes of professionalism. Honor/Integrity was the most valued trait and Excellence/Autonomy was the lowest valued trait of medical professionalism.

## Introduction

Medical professionalism is one of the core competencies of physicians ( 1 , 2 ). It, along with the expectation of professional competency, is the relationship of trust between the physicians and society ( 3 - 6 ). Professionalism has been the focus of attention in medical education during the last two decades along with its challenges of teaching, learning and assessing ( 7 ). The attitude of young medical graduates is a source of concern for all medical educationists. This highlights the need for formal teaching and assessment of medical professionalism ( 8 ). The conduct of doctors rather than their competency is the reason for most of the complaints against doctors ( 8 , 9 ). It is suggested that professionalism should be a part of training of medical students in order to minimize any form of professional misconduct in future practice ( 9 - 11 ). Such trainings are often an implicit part of curricula ( 12 , 13 ). However, the emphasis on formal training of medical professionalism to achieve it as a competency has only intensified recently ( 14 , 15 ). To achieve this goal, different teaching modalities are being used for formal teaching including case scenarios, reflection, class lectures, small group discussions, bedside teaching and use of videos, etc. ( 15 - 17 ). The assessment of medical professionalism is, however, a challenge. There is no single accepted tool of assessment. Various assessment tools used are self-evaluation, practical examinations, structured exams, incident reporting and developing portfolios ( 6 , 10 , 18 ). The self-assessment tools provide an insight about the individuals’ perception of different domains of medical professionalism. It can be used to identify the strengths and weaknesses of individuals.

The medical students learn professionalism throughout their undergraduate years both formally and informally. As freshmen, medical students learn the theoretical concepts and philosophy of professional conduct. The faculty of the medical school also acts as their role models. The clinical placements play a significant role in learning professionalism from the faculty ( 15 ). In order to assess various traits of professionalism in the students and faculty of SMDC, we decided to use a self-assessment tool. The Learner’s Attitude of Medical Professionalism Scale (LAMPS) devised and validated by M Al-Eraki in 2013 was used ( 19 ). The students of the First and Final Year MBBS and faculty members were assessed and compared. The purpose of this study was to know the perceived gaps in various domains of medical professionalism. This would be helpful in modifying the curriculum of the medical college, if required. 

## Methods

### Settings

The study was conducted at Shalamar Medical and Dental College, Lahore from June 2018 to Dec 2018 after approval from the Institutional Review Board (IRB) of SMDC.

### Subjects

First Year and Final Year students of SMDC at the end of their academic year were enrolled in the study, using non-probability convenience sampling. Sample size was calculated by the formula presented by Cochran and David. By using a questionnaire response in five-point scale, a minimum sample size of 119 was calculated, using a level of significance of 5%and a margin of error of 3%. In view of unevenness of the study and expected nonresponse, 200 subjects were recruited to achieve the research objectives ( 20 ). LAMPS questionnaire was distributed among 100 students from each class who agreed to participate in the research voluntarily. Each class was approached by the researchers when the students were in their lecture halls, just after finishing their lectures. They were all informed about the purpose of the study and their voluntariness to participation in the study. They were handed questionnaires and consent forms. Only the students willing to participate in the study returned the anonymously filled out questionnaires. A total of 50 faculty members from Basic and Clinical Sciences agreed to participate. The students and faculty members were briefed about the research.

### Methods

This is a cross-sectional survey. Non-probability convenience sampling was used. Sample size was calculated by applying the formula of Cochran and David. The level of significance was kept at 5%, and the margin of error was 3 percent ( 20 ). LAMPS was used as the study instrument after taking permission from Al-Eraki who devised and validated it in 2003 in the Arabian context ( 19 ). This is a self-assessed perception of the attitude in different domains of professionalism. Duty Accountability, Excellence/ Autonomy, Honor/Integrity, Altruism, and Respect were assessed by 28 items. Seven items were for Duty/Accountability, 6 items for Excellence/Autonomy and 5 items each for Honor/Integrity, Altruism and Respect. In each item, the participants were asked about their behavioral response to a given scenario. The responses were on a Likert Scale where [1] represented strong disagreement and [5] represented strong agreement. However, the reverse was true in the case of negative statements where [1] represented strong agreement and [5] represented strong disagreement. The overall reliability of LAMPS by Cronbach’s alpha was 0.7. The reliability of individual domains, i.e. Respect, Excellence/Autonomy, Altruism, Duty/Accountability and Honor/Integrity was 0.57, 0.48, 0.42, 0.57 and 0.43, respectively. A reliability ranging from 0.3 to 0.6 was considered to be moderate. LAMPS has been derived from the definition of professionalism given by American Board of Internal Medicine. Its content validity was confirmed by 32 experts in medical education after piloting with more than 300 respondants ( 19 ). The instrument was used in its original form and English language. The students were given the questionnaire along with the consent form in their classrooms. They were given half an hour to respond, and then the forms were collected. The faculty were given two weeks to respond. 

### Inclusion Criteria

First and Final Year MBBS students and faculty members who consented to participate were included in the study.

### Exclusion Criteria

Incomplete questionnaires were excluded from the study.

### Statistical Analysis

The data were analyzed using SPSS 24. The means for each domain were calculated and compared by using t-test. The comparison was made between the students of First Year and Final Year, and between the students of Final Year and Faculty. P value less than 0.05 was considered as significant.

### Ethical Consideration

The study proposal was approved by an OHRP (Office of human Research Protection) registered Institutional review Board of Shalamar Medical & Dental College, Lahore (SMDC-IRB-068, dated on 7th June, 2018). The study was initiated after the issuance if approved letter. The data was anonymously collected and was kept confidential by the researcher.

## Results

204 study participants in this study included 166 students and 38 faculty members. Out of 166 students who completed the survey, 88 were from the First year and 78 from the Final year groups. Out of 50 faculty members, 38 of them completely filled the survey. Hence, the response rate was 88%, 78% and 76% for the first year, final year and faculty, respectively. The students and faculty members, who had not filled the questionnaire completely, were excluded from the study. The domains of professionalism that were assessed included Duty/Accountability, Excellence/Autonomy, Honor/ Integrity, Altruism and Respect. 

**Figure 1:**
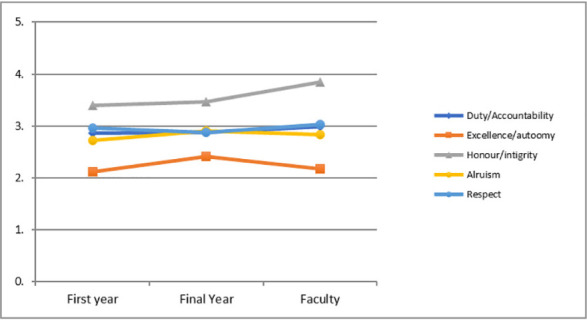
Comparison of Means of Perception Scores of Professionalism among First Year & Final Year Students and Faculty of Shalamar Medical & Dental College (SMDC) by using Learner’s Attitude of Medical Professionalism Scale (LAMPS)

Table 1 shows the demographic data of the participants, including their mean age and gender distribution. Table 2 shows the comparison of different domains of professionalism between the First year and Final year. Honor/Integrity was found to be the most frequently selected attitude of medical professionalism, whereas Excellence/Autonomy remained the least selected domain by the First year and Final year MBBS students. The p values of the domains of Excellence/Autonomy and Altruism were found to be statistically significant, whereas p values of the rest of the domains were not statistically significant. Table 3 shows comparison of different domains of professionalism between the Final year students and faculty. The faculty of SMDC also rated Honor/Integrity as the most frequently valued attribute of medical professionalism. Excellence/Autonomy remained the least frequently valued domain by the Final year MBBS students as well as the faculty members. The p values of the domains of Excellence/Autonomy and Honor/Integrity were found to be statistically significant, whereas those of the rest of the domains were not statistically significant. The comparison of the means of domains of professionalism among the First year, Final year students and faculty of SMDC by using LAMPS is shown in Figure 1. Honor/Integrity was the most frequently valued trait and Excellence/Autonomy was the lowest scored domain of medical professionalism by all three groups. Honor/Integrity was the domain with the highest score in the faculty and lowest among the first year students. The reverse was found to be true for Excellence/Autonomy, whereas the rest of the domains did not show variability.

**Table 1:** Demographic data including mean age and gender distribution among the First Year & Final Year Students and Faculty of SMDC

	First Year (n=88)	Final Year (n=78)	Faculty (n=38)
Age (Average)	18.9	24.19	51.3
Males	41(47.10%)	36(47%)	22(58%)
Females*	47(52.9%)	42(53%)	16(42%)

* SMDC: Shalamar Medical & Dental College

**Table 2:** Comparison of the Perceptions of Professionalism between the First Year and Final Year Students of SMDC by using LAMPS

Domains	First Year (Mean±SD)	Final Year (Mean±SD)	P
Duty/Accountability	2.86±1.38	2.88± 1.34	0.858
Excellence/Autonomy	2.12±1.02	2.42 ±1.16	0.000
Honor/Integrity	3.47±1.34	3.40±1.26	0.459
Altruism	2.72±1.28	2.90±1.24	0.041
Respect*	2.96±1.54	2.88±1.43	0.449

* SMDC: Shalamar Medical & Dental College,

* LAMP: Learner’s Attitude of Medical Professionalism Scale

**Table 3:** Comparison of Perceptions of Professionalism between Final Year Students and Faculty of SMDC by using LAMPS

Domains	Final Year (Mean±SD)	Faculty (Mean±SD)	P
Duty/Accountability	2.88± 1.34	2.99±1.43	0.264
Excellence/Autonomy	2.42 ±1.16	2.17±1.26	0.009
Honor/Integrity	3.40±1.26	3.84±1.23	0.000
Altruism	2.90±1.24	2.83±1.34	0.546
Respect *	2.88±1.43	2.83±1.34	0.400

* SMDC: Shalamar Medical & Dental College,

* LAMP: Learner’s Attitude of Medical Professionalism Scale

## Discussion

Medical professionalism is a set of values, beliefs, behaviors, and attitudes, which a society expects from a doctor ( 3 - 6 ). Professionalism may be looked at differently by medical students and faculty. This makes it necessary to have a standardized curriculum for undergraduate medical students ( 21 , 22 ). Before embarking on such an exercise, it is worthwhile to understand the perceptions of medical students and faculty members about certain values of medical professionalism. This study was performed to identify the perception gaps in various domains of medical professionalism, thereby elucidating where teaching of medical professionalism should be focused. This identified the strengths and weaknesses about perception of different domains of medical professionalism among the students and faculty ( 19 ). 

Medical professionalism does not have a single standardized definition because of cultural and contextual differences ( 23 - 25 ). However, there are certain domains/traits of medical professionalism that have been incorporated in the definition of medical professionalism. American Board of Internal Medicine (ABIM) has described altruism, accountability, duty, excellence, integrity, honor, and respect as six components of medical professionalism ( 26 ). These components were utilized by Al-Eraki to develop and validate the professionalism assessment scale in the Arabian context and named this tool as Learner’s Attitude of Medical Professionalism Scale (LAMPS). This is a self-assessment tool about the behavioral perception of different domains of professionalism. Duty/Accountability, Excellence/Autonomy, Honor/Integrity, Altruism and Respect are assessed. Honor/Integrity is referred to as honesty, truthfulness to the patients and colleagues assessed by five items in LAMPS. Excellence/Autonomy, assessed by six items is the updated evidence-based knowledge and adherence to the ultimate principles of ethics. Altruism, selflessness for patients or keeping best interest of patients foremost are assessed by five item questions. Duty/Accountability, assessed by seven questions in LAMPS, is being responsible, dutiful, and answerable to peers, patients, as well as society ( 19 ). Similarly, five items are used to assess Respect. In each item, different scenarios are given in order to assess the perception about the attitude regarding medical professionalism. The Arabian context is similar to that of our country because of the same religion and similar cultural values. LAMPS is a validated instrument, so it was used in our research to assess and compare the attitude of medical professionalism of undergraduates (First year and Final year) and faculty of SMDC, Lahore ( 27 ).

Honor/Integrity was found to be the most valued attitude of medical professionalism, whereas Excellence/Autonomy remained the lowest valued domain by the First year and Final year MBBS students. Results reported by Vikram Jha also suggest integrity as one of the most valued conceptual components of professionalism by students and professionals ( 28 ). This study, however, used qualitative methods and included five different groups of students and professionals, unlike our study. He also concluded that the term professionalism is complicated and contextual.

The comparison of the perception of professional attitude between the First year and Final year students at SMDC showed a statistical difference in the mean score for the domains of Excellence/Autonomy and Altruism. Final year students valued Altruism and Excellence/Autonomy more than other attributes. This difference in attitudes may be explained by limited exposure to clinical settings and patients for the first-year students. Final year students experience the clinical environment and, hence, have different perceptions of medical professionalism. Even though the first-year medical students do not have a direct encounter with patients, medical professionalism is a part of the formal as well as informal curriculum. At SMDC, professionalism in incorporated in the subject of “Behavioral Sciences” as per advice of Pakistan Medical and Dental Council (PMDC) and University of Health Sciences (UHS). The students at SMDC are also taught “Bioethics” as a formal subject that also incorporates various components of medical professionalism. Class lectures, small group discussions, clinical scenarios, case scenarios, and reflective practices are used for formal teaching of medical professionalism. Thus, it would not be wrong to assume that the First-year medical students at SMDC have some insight into medical professionalism. As documented by S Mine, first-year medical students in Turkey identified geniality, ability to communicate well, humaneness and benevolence as the most important traits of a ‘good physician’. Al-Eraki reported a better mean score for pre-clinical students compared to clinical students in all domains of medical professionalism ( 29 ). The decline in the perception about attitudes regarding medical professionalism from pre-clinical to clinical medical students raises a number of questions about the factors affecting medical professionalism in future physicians. This also emphasizes the importance of teaching medical professionalism as a longitudinal theme in a formal curriculum of medical schools ( 30 ).

The remaining domains of professionalism, however, showed almost similar mean scores. The domains of Duty/Accountability, Honor/Integrity and Respect have been perceived well by the end of the first year, as reflected in our research. This highlights the significance of formal training of medical professionalism in the preclinical years. 

The faculty of SMDC also rated Honor/Integrity as the most valued attribute of medical professionalism. Excellence/Autonomy remained the lowest valued domain by Final year MBBS students as well as faculty. Similarly, the students and faculty of medical college rated honesty as the most valuable domain of professionalism ( 26 , 28 ).

A comparison of means in the domains of professionalism between the Final year students and faculty revealed a significant variation in Honor/Integrity, which was higher in the faculty. These findings are similar to those of the research done in the Arabian context by Al-Eraki who found Honor/Integrity as the domain with highest score when compared with other domains of professionalism in undergraduate students ( 30 ). Although Al-Eraki found only Honor/Integrity to be better in the faculty as compared to the students, all of the other domains of professionalism were better in students. This is different from our study as Excellence/Autonomy was the domain with a higher mean score in the Final year students, as compared to the faculty. Jahan’s study comparing medical professionalism among medical students and faculty in Oman revealed a significant difference in the professional attitudes of the two. The students and faculty agreed in the domains of professionalism like up-to-date knowledge, respect for others, teamwork, and ethical thinking; however, there was a statistically significant difference for communication skills, self-management, and risk management ( 31 ). This research also reflects the perception of students and faculty about their attitude regarding medical professionalism. However, knowledge about medical professionalism was not found to be different among students and faculty in Iran by M S Farshad ( 5 ). Therefore, self -perception of the faculty and student about the attitudes/behaviors of medical professionalism did vary and this signifies the need for training and development of medical professionalism throughout medical school ( 10 ).

Results showed that the lowest mean score was in the domain of Excellence/Autonomy with a mean of 2.2 to 2.8. The highest mean score was in Honor/Integrity with a mean 3.5 to 4.3. Similar results were found by R Munazzah ( 32 ). However, Al-Eraki reported that the domain of Respect to others had the highest score and Honor/Integrity had the lowest; this is not consistent with the findings of our study ( 19 ). Over time, the importance of Honor/Integrity continues to rise as one is involved in the practice of medicine. This may reflect the maturity of the physicians as they gain experience. 

The domain of Excellence/Autonomy received the lowest mean score which increased by the end of medical school and then suddenly declined afterwards. This may be attributed to limited interaction with patients for the first-year students. The decline in the attitude of the faculty members might be due to heavy workload like academic activities, clinical activities, administrative engagements, family commitments, non-conducive work environment, etc. Al-Eraki ascribed this change in attitudes to a lack of interest and decline in the professional attitudes of physicians ( 30 ). 

The mean scores of the other three domains did not show any variance. The students entering a medical school bring along with them their personal and family values. Their transformation to a physician begins with virtues of a healer, leader, and communicator. Hence, the first year of medical school is the time to start formal teaching of medical professionalism. The training should emphasize the professional qualities throughout the medical school, so that these are inculcated in their characters as they graduate ( 33 ).

Role modeling of faculty/teachers by medical students is advocated as one of the most influential methodologies of teaching medical professionalism. The same is true in our study, as Honor/Integrity is the most valued domain by teachers as well as students. This was true for Excellence/Autonomy which was the domain with the lowest score by faculty and students ( 34 , 35 ). Thus, continuous professional development in the form of training and workshops for the faculty is essential to keep them updated on the requirements of medical professionalism.

## Limitations

This is a cross-sectional study on a single institution, so the results cannot be generalized. This study is based on perceptions of medical students and faculty, as reported by them. Therefore, some reporting bias may be present. Further qualitative and longitudinal studies are required to understand the reasons of the varied perceptions. This self-administered questionnaire reflects the self-perception of attitudes about different domains of medical professionalism and does not reflect the actual professional behavior.

## Recommendations and Suggestions

Formal training of medical professionalism should start from the first year MBBS, so that the students have an insight about medical professionalism and display a behavior worthy of a medical professional. This is a continuous learning process and must continue throughout medical school. The trainings should continue even after graduation as practicing physicians and teachers have a significant role in professional development of young graduates and students. Institutions have a professional and moral responsibility to develop the resources for such continuous professional development. 

## Conclusion

Medical students and faculty members have significantly different views of certain attributes of professionalism. The perceptions of the first year and final year medical students differ about Excellence/Autonomy and Altruism. All the attributes of medical professionalism are an important part of medical education that must be reflected in medical professionals. It is imperative for the field of medical education to focus on these attributes. The faculty, institutions, and all healthcare professionals share the responsibility to not only demonstrate, but also inculcate these attributes among their students and junior colleagues. Formal teaching and learning of medical professionalism should begin from the first year of medical school.
